# Evaluation of the Plasma Micro RNA Expression Levels in Secondary Hemophagocytic Lymphohistiocytosis

**DOI:** 10.4084/MJHID.2013.066

**Published:** 2013-11-04

**Authors:** Ali Bay, Enes Coskun, Serdar Oztuzcu, Sercan Ergun, Fatih Yilmaz, Elif Aktekin

**Affiliations:** 1 Gaziantep University Department of Pediatrics Division of Pediatric Hematology Gaziantep/Turkey; 2 Gaziantep University Department of Pediatrics, Gaziantep/Turkey; 3 Gaziantep University Department of Medical Biology, Gaziantep/Turkey

## Abstract

**Background:**

Hemophagocytic lymphohistiocytosis (HLH) is a life threatening hyper inflammatory disease. Micro RNAs (miRNA) are about 22 nucleotide-long, small RNAs encoded with genes, and they have regulatory functions in immune response.

**Objective:**

To determine the miRNA expression levels of 11 secondary HLH patients, we evaluated the associations of miRNA levels with pathogenesis, clinical presentation, and prognosis of the disease.

**Patients and Methods:**

Patients who were diagnosed with secondary HLH from January 2011 to December 2012 were included in this study. We profiled the expressions of 379 miRNAs in plasma of both HLH patients and healthy controls. Patients were evaluated regarding with age, clinical findings, miRNA expresions, laboratory data, treatment, and prognosis, by using descriptive statistics.

**Results:**

A total of 11 secondary HLH patients and 11 healthy children were included in this study. miR-205-5p was expressed in all case and controls and expression level of miR-205-5p was found 6.21 fold higher than control group (p=0.01). We detected the second highest expression percent in miR-194-5p with 81% of cases and controls. Expression level of miR-194-5p was found to have 163 fold higher than controls (p= 0.009). miR-30c-5p showed 77% expression percent in cases and controls together. The expression level of this miRNA was detected 9 fold decreased in HLH patients compared to healthy children (p= 0.031).

**Conclusion:**

We showed that miR-205-5p, miR-194-5p and miR-30c-5p could be useful plasma biomarkers for HLH. Further research is needed in larger and homogenous study groups, especially for these miRNAs as biomarkers for HLH.

## Introduction

Hemophagocytic lymphohistiocytosis (HLH) is a life threatening hyper inflammatory disease caused by an uncontrolled and dysfunctional immune response. It is characterized by activation and massive proliferation of T cells and macrophages, leading to marked hyper cytokinemia.^1^ HLH frequently develops in patients with underlying genetic disease (primary or familial HLH), but can also occur secondary to infection, malignancy, metabolic or autoimmune diseases.^2^ Patients manifesting with HLH in the absence of a disease-causing mutation in the known genes and without strong indications for a genetic predisposition, such as familial disease or recurrent episodes of HLH, are currently classified as secondary HLH. Although four genes, accounting for over 90% of familial cases, have been identified, final classification of a patient as suffering from ‘secondary’ HLH must remain preliminary.^1^,^3^

HLH is characterized by highly activated lymphocytes and macrophages that infiltrate tissues and produce large amounts of proinflammatory cytokines. Defects in natural killer (NK) cell- and cytotoxic T cell (CTL)-mediated cytotoxicity may impair elimination and control of intracellular pathogens, leading to prolonged and enhanced stimulation of immune cells and subsequent immunopathology.^4^ Notably, the cytotoxic effector response not only targets infected cells, but also antigen-presenting cells (APC). Elimination of APC in the context of an effective immune response is an important negative feedback to curtail the immune response. Impaired cytotoxicity disrupts this negative feedback and leads to continued stimulation of activated NK cells and CTL secreting large amounts of cytokines, in particular c-interferon, which is a potent macrophage-activating stimulus. This disturbance of immune homeostasis can also be caused by infections, autoimmune or neoplastic disease in the absence of a genetic defect of lymphocyte cytotoxicity.^5^ The pathogenesis of secondary HLH is not well understood. CTL degranulation and cytotoxicity are not impaired in most cases. Nevertheless, the balance between APC activation and CTL-mediated control may be disrupted through increased APC activation.^6^

Micro RNAs (miRNA) are about 22 nucleotide-long, small RNAs encoded with genes, and they have regulatory functions in protein synthesis by inhibition of translation and/or increasing the degradation of mRNA after binding to complementary region of related mRNA.^7^–^10^ They involve in many crucial biological events hereby any dysfunction in its regulation may cause many disorders including autoimmune diseases.^11^ The roles of miRNAs in inflammation, T-cell, and NK cell function have been demonstrated in several studies.^12^ From this point of view we aimed to investigate the miRNAs related to HLH that is a disease caused by an uncontrolled and dysfunctional immune response.

We measured the plasma miRNA expression levels of 11 secondary HLH patients who were followed up in our clinic. We also evaluated the associations of miRNA levels with pathogenesis, clinical presentation, and prognosis of the disease. This is the first research in literature about this subject based on our knowledge.

## Patients and Methods

We diagnosed 16 patients as HLH from January 2011 to December 2012 according to Diagnostic Guidelines for HLH 2004.^13^ The patients were classified as having primary HLH if there was a severe clinical presentation without a proven or suspected infection history or metabolic disease and at least one of the following criteria: family history or parental consanguinity, severe clinical presentation with central nervous system involvement, or persistence or recurrence of HLH. Due to the fact that genetic mutation was detected in 5 of them upon genetic analyzes, they were diagnosed as primary HLH. Therefore, the other 11 patients in our study were classified with secondary HLH because they had suspected or proven infection, no family history or parental consanguinity, had no central nervous system involvement, and had no prior history of HLH. All patients fulfilled at least five cardinal criteria of HLH at the time of diagnosis, including fever, hepatosplenomegaly, bicytopenia and/or pancytopenia, hypertriglyceridemia and/or hypofibrinogenemia, hyperferritinemia, and hemophagocytosis in the bone marrow. Patients were evaluated regarding with age, clinical findings, miRNA expresions, laboratory data, treatment, and prognosis, using by descriptive statistics.

### miRNA isolation

Blood samples were taken at diagnosis of HLH and before the treatment. Whole blood samples taken into tubes with EDTA from HLH and control subjects were centrifuged at 4000 rpm. Then, total RNA including miRNAs were extracted from plasma by using miRNeasy Mini Kit (Qiagen, GmbH, Hilden, Germany, Cat. No.:217004).

### cDNA conversion

Isolated RNA samples were converted to complementary DNA (cDNA) using TaqManmiRNA Reverse Transcription Kit (Life Technologies, Foster City, CA) in 384 well Thermal Cycler (BioEr, China). MegaPlex Human RT Primer Pool A and B were used as Reverse Transcription primers.

### PreAmplification

Prior to qRT-PCR reactions, the cDNA samples were preamplified using TaqManPreAmp Master Mix (Life Technologies, Foster City, CA). The preamplification protocol was as follows: 95°C for 10 min, 55°C for 2 min., 72°C for 2 min. and as cycling step 95°C for 15 sec and 60°C for 4 min, for 14 cycles. The preamplified cDNA samples were kept at −20°C for further analysis.

### Quantitative Real-Time Polymerase Chain Reaction (qRT-PCR)

QRT-PCR reactions were performed with a high throughput instrument (BioMark; Fluidigm, San Francisco, CA). Preamplified cDNA samples were mixed with TaqMan Universal PCR Master Mix (No AmpErase UNG, Applied Biosystems, Foster City, CA) and Sample Loading Reagent (Fluidigm, San Francisco, CA) and pipetted into sample inlets of the Dynamic Array 96.96 chips (Fluidigm, San Francisco, CA). QRT-PCR reactions were performed in the BioMark Real-Time PCR system using the following protocol: 10 min at 95°C, 15 sec at 95°C and 1 min at 60°C for 30 cycles." was changed as "QRT-PCR reactions were performed in BioMark™ HD Dynamic Array Real Time PCR system (Fluidigm, San Francisco, CA, USA) with Megaplex™ Primer Pools, Human Pools A v2.1 (Applied Biosystems®, Foster City, CA) using the following protocol: 10 min at 95°C, 15 sec at 95°C and 1 min at 60°C for 30 cycles. Therefore, all analyses were performed in duplicates

### Statistical Methods

SPSS 14 statistical software was used for analysis. miR-34a-5p was used for normalization of individual miRNA expression levels. Fold change is calculated by formula 2^−ΔΔCt^.

ΔΔCt: (normalized mean cq value of HLH) − (normalized cq value of controls). Decrease in expression showed as negative fold change.

Student t test and Mann-Whitney U test was used and p<0.05 was evaluated as statistically important.

## Results

A total of 11 secondary HLH patients and 11 healthy children were included in this study. Five of HLH patients were male and six of them were female. The average age of HLH patients and healthy children were 103±17 and 98±37 months, respectively. The age and gender distribution between patients and control group was the same. The clinical and laboratory data of the patients group was revealed in table 1.

We profiled the expressions of 379 miRNAs in plasma of both HLH patients and healthy controls. We compared the expression levels of miRNAs between these groups. Four miRNAs (miR-34a-5p, miR-205-5p, miR-302c3p, and miR-483-5p) were consistinly expressed in both groups. We compared raw quantification cycle (Cq) values between HLH and control group to determine a housekeeping gene for normalization. Among these four candidate genes, miR-34a-5p showed no difference between groups and its standard deviation was the lowest; therefore this miRNA was chosen as housekeeping gene for this study (T**able 2**).

miR-205-5p was expressed in all case and controls. The expression level of miR-205-5p was found 6,21 fold higher than control group (p=0.01). We detected the second highest expression percent in miR-194-5p with 81% of cases and controls. Expression level of miR-194-5p was found to have 163 fold higher than that of controls (p= 0.009). miR-30c-5p showed 77% expression percent in cases and controls together. The expression level of this miRNA was detected 9 fold decreased in HLH patients when compared to that of healthy children (p= 0.031). The calculated fold change was 0.1 that is expression reduced as 9 fold. Because of low expression levels of miR-192-5p and miR-618, statistical analyses of these miRNAs were not found noteworthy (Table 3).

In secondary HLH patients, it was investigated whether there was a positive or negative correlation between miRNA expression and age, sex, concurrent disease, and laboratory values. A weak positive correlation between miR-192-5p and age (p= 0.029) and, a weak negative correlation between miR-618 and hemoglobin level (p= 0.027) was found. Because of small number of population, these detected correlations are not valuable.

## Discussion

HLH is a genetically heterogeneous disorder characterized by a hyper-inflammatory syndrome with fever, hepatosplenomegaly, cytopenia and sometimes central nervous system involvement.^14^ Secondary HLH is still an important, elusive and misdiagnosed condition in spite of developed knowledge. Improving evidence shows that small noncoding gene products, miRNAs, negatively regulate gene expression post transcriptionally.^15^,^16^ miRNAs are included in many of important activities in biological life cycle, including cell proliferation, differentiation, and apoptosis and play a significant role in inflammatory diseases, in the vascular system and diabetes.^17^–^19^ Array analysis showed that a huge number of miRNA are potentially differentially regulated between different disease states and healthy controls in ovarian cancer.^11^ Herein, we have shown that five of miRNAs (miR-205-5p, miR-194-5p, miR-30c-5p, miR-192-5p, miR-618), which were found to possibly target genes related to inflammation based on the Target Scan and MiRanda databases, are differentially regulated during hyper-inflammation realized in HLH patients (Table 4).

The transcription factor E2F1 is one of the key proteins in the regulation of the G1/S phase transition, hence it acts as a critical regulator of cell survival and proliferation^20^ Previous publications identified E2F1 as a transcription factor involved in the regulation of inflammatory response to Toll-like receptor ligands.^21^ Furthermore, cell cycle-related protein E2F5 is chronically up regulated upon chronic inflammation.^22^ Therefore, with substantially increased expression, miR-205-5p targeting E2F1 and E2F5 might show the pathophysiology of HLH, as an inflammatory disease, with its effect on E2F1 and E2F5

Transcriptional co activator p300 phosphorylation at Ser89 by AMPK is critical for the therapeutic effect of AMPK and may be a potential target for pharmaceutical intervention in inflammatory diseases.^23^ Also, nuclear factor kappaB (NF-KB) plays a critical role in the transcriptional regulation of genes involved in inflammation and cell survival. PARP-1 and p300 synergistically co activated NF-KB-dependent gene expression in response to TNF alpha and LPS.^24^ miR-195-5p up regulated in HLH patients when compared to healthy subjects. Thus, upon these two remarkable contributions of p300, miR-195-5p expression level can reflect the pathophysiology of HLH in that miR-195-5p is closely associated with the expression of p300.

MUC17 is substantially expressed in normal colonocytes both in the proximal and distal colon, and that the expression of this protein is remarkably reduced in inflammatory and neoplastic conditions. These results suggest that MUC17 takes a pathogenetic role in inflammatory and neoplastic conditions of the colon.^25^ According to our findings, expression of miR-30c-5p targeting MUC17 was reduced. From that point, we can predict that normally miR-30c-5p is upregulated and its effect on upstream cascade of MUC17 expression provides MUC17 upregulation. However, in inflammatory and neoplastic conditions, downregulation of miR-30c-5p expression causes decreasing in expression of MUC17 via upstream cascade of expression of it. Therefore, this relationship explains the association of HLH with downregulation of miR-30c-5p.

The expression percents of both miR-192-5p and miR-618 were very low so statistical analyses of these miRNAs were not meaningful to comment on.

In a study using the human NKL cell line, stimulation through NKG2D led to down regulation of a number of miRNAs.^26^ One of these miRNAs, miR-30c was shown to target HMBOX1, an inhibitory transcription factor of cytokine secretion. Overexpression or inhibition of miR-30c resulted in altered killing against two hepatoma cell lines, again with caveat that additional validation using primary NK cells is needed. Moreover, two studies sequenced the small RNA compartments of resting and IFN-alpha^27^ or IL-2/-15/-21^28^ activated human peripheral blood CD56+CD3− NK cells. While the majority of significantly changed miRNAs in IFN-alpha treated NK cells were down regulated, most miRNAs in IL-2/-15/-21 treated cells were upregulated. These differences may reflect miRNA changes that are specific to the mode of NK cell stimulation or the time frame of measurement.^29^ In second of these studies, one of these regulatory miRNAs is miR-192. Because miR-192 is one of the top expressed miRNAs in this study and it is thought that it has regulatory roles in NK cell-mediated killing. These facts on the effects of two of our related miRNAs on NK cells, miR-30c and miR-192 provides valuable clues for the further elucidation of microRNA regulation in human NK cell activation and may have a great potential in NK cell immunotherapy.

Secondary HLH is an impossible diagnosis to confirm outside the setting of proven autoimmune disease or malignancy. Although, despite attempts to differentiate primary from secondary HLH, the symptomatic presentations are highly overlapping. Patients with HLH in the context of an underlying genetic disease frequently suffer from relapses during treatment or experience additional episodes of HLH with a high risk of a lethal outcome. This can only be prevented by correction of the defective immune system by haematopoetic stem cell transplantation. All of our patients have alived without relapses although they have been treated with short-time chemotherapy.

Secondary HLH is an etiologically heterogeneous entity. Given the diverse biological, clinical signs and laboratory abnormalities; secondary HLH is often misdiagnosed or altogether unrecognized, resulting in high mortality rates. We investigated miRNA expressions as possible diagnostic or prognostic agent in secondary HLH patients. In fact, this is the first study dealing with miRNAs in HLH patient group. In our study, no correlation was found between secondary HLH patient laboratory values and miRNA expression levels. This may be resulted from low number of patients and heterogeneity of study group.

There are some limitations in our study. First of all, our study population is so small. If we could collect much more HLH patients, our results would be validated strongly. However, HLH is a rare disease so it is very difficult to collect enough number of patients. Our second limitation is the characterics of control group. If we could find patients with inflammatory diseases other than HLH as control group, our results would be more valuable.

## Conclusion

to date, very little data exists regarding the expression of the miRNAs to investigate HLH. In this study, we showed that miR-205-5p, miR-194-5p and miR-30c-5p can be useful markers for HLH. Combination of these newly discovered biomarkers would be useful for the understanding of patient’s status with HLH. Further research is needed to determine the specific role of other inflammatory related miRNAs in the pathogenesis of HLH in larger and homogenous study groups as well as use of these miRNAs as biomarkers for HLH.

## Figures and Tables

**Table 1:** Patient characteristics

Patient no	1	2	3	4	5	6	7	8	9	10	11
**Gender**	M	F	M	F	F	F	F	M	F	M	M
**Age (month)**	5	27	57	63	91	136	137	149	155	156	158
**Concurrent disease**	Hepatitis A		Chronic granulamatous disease	Brain abcess due to TOFF	Chronic renal failure	Myelodysplastic syndrome	Brucellosis	Thyphoid fever	Chronic renal failure	İnfectious Mononucleosis	Hepatitis A
**Detected agent**	Hepatitis A	Ebstein Barr Virus	Staphylococcus	Staphylococcus			Brucella	S.tyhphi		Ebstein Barr Virus	Hepatitis A
**Hemoglobin (g/dl)**	7	6	7,4	8,3	8,1	9	9,6	9,8	5,4	7,6	9,2
**WBC (/mm^3^)**	2200	1600	2400	2900	1800	700	1800	3300	2300	1900	1600
**Platelet (/mm^3^)**	96000	88000	74000	21000	54000	61000	98000	50000	18000	46000	39000
**Triglyceride (mg/dl)**	302	245	203	445	92	275	474	188	481	134	189
**Fibrinogen (mg/dl)**	128	110	275	77	327	624	320	213	86	146	177
**Ferritin (ng/ml)**	38773	4850	9131	24922	27417	3525	12141	3712	40000	5408	10392
**ALT (IU/L)**	150	120	143	30	71	29	121	96	13	124	56
**AST (IU/L)**	399	312	691	179	124	15	169	79	173	163	281
**Hemophagocytosis in bone marrow**	Positive	Positive	Positive	Positive	Positive	Positive	Positive	Positive	Positive	Positive	Positive
**Prolonged fever**	Positive	Positive	Positive	Positive	Positive	Positive	Positive	Positive	Positive	Positive	Positive
**Hepatosplenomegaly**	Positive	Positive	Positive	Positive	Positive	Positive	Positive	Positive	Positive	Positive	Positive
**Outcome**	Live	Live	Live	Live	Live	Live	Live	Live	Live	Live	Live

**Table 2:** List of completely expressed miRNAs

	Number of patient	Mean	Std. Deviation	
**miR-34a-5p**	22	12.7378	1,7634	Housekeeping gene
**miR-205-5p**	22	13.8944	2,05745	
**miR-302c-3p**	22	12.6458	2,82873	
**miR-483-5p**	22	13.6035	5,99616	

**Table 3:** List of miRNAs that have significant fold change

miRNA	HLH	Control	Expression %	Foldchange	P
miR-205-5p	11	11	100.00	6.21	0.01
miR-194-5p	9	9	81.82	163.36	0.009
miR-30c-5p	7	10	77.27	0.10	0.03
miR-192-5p	3	6	40.91	926,02	0.043
miR-618	6	3	40.91	610359.62	0.055

**Table 4:** Genes related with inflammatory targeted by miRNAs having meaningful expression change in HLH (according to TargetScan and MiRanda databases)

	Targeted gene
**miR-205-5p**	E2F1
**miR-194-5p**	P300
**miR-30c-5p**	MUC17, HMBOX1
